# Gelatin-Based Liver Phantoms for Training Purposes: A Cookbook Approach

**DOI:** 10.3390/jcm13123440

**Published:** 2024-06-12

**Authors:** Radu Claudiu Elisei, Florin Graur, Amir Szold, Andreas Melzer, Sever Cãlin Moldovan, Mihai Motrescu, Emil Moiş, Cãlin Popa, Doina Pîsla, Cãlin Vaida, Tiberiu Tudor, Adrian Coţe, Nadim Al-Hajjar

**Affiliations:** 1 Department of Surgery, University of Medicine and Pharmacy “Iuliu Hatieganu”, 400012 Cluj-Napoca, Romania; radu_elisei@yahoo.com (R.C.E.); drmoisemil@gmail.com (E.M.); calinp2003@yahoo.com (C.P.); na_hajjar@yahoo.com (N.A.-H.); 2 Emergency Clinical County Hospital, 420016 Bistrita, Romania; severcalinmoldovan@gmail.com (S.C.M.); mimotrescu@gmail.com (M.M.); drtiberiutudor@gmail.com (T.T.); 3 Regional Institute of Gastroenterology and Hepathology “Dr. Octavian Fodor”, 400394 Cluj-Napoca, Romania; 4 Assia Medical, Assuta Medical Center, Tel Aviv 6971028, Israel; amikisz@gmail.com; 5 ICCAS Insitute of Computer Assisted Surgery, University Leipzig, 04109 Leipzig, Germany; a.melzer@dundee.ac.uk; 6 IMSAT Insitute for Medical Science and Technology, University Dundee, Dundee DD1 4HN, UK; 7 CESTER Department, Faculty of Industrial Engineering, Robotics and Production Management, Technical University of Cluj-Napoca, 400114 Cluj-Napoca, Romania; doina.pisla@mep.utcluj.ro (D.P.); calin.vaida@mep.utcluj.ro (C.V.); 8 Emergency County Hospital, 410159 Oradea, Romania; adrian.cote@gmail.com

**Keywords:** gelatin-based phantom, liver mold, liver phantom, 3D printing, liver surgery, liver biopsy, training, ultrasound-guided

## Abstract

**Background**: Patients with liver pathology benefit from image-guided interventions. Training for interventional procedures is recommended to be performed on liver phantoms until a basic proficiency is reached. In the last 40 years, several attempts have been made to develop materials to mimic the imaging characteristics of the human liver in order to create liver phantoms. There is still a lack of accessible, reproducible and cost-effective soft liver phantoms for image-guided procedure training. **Methods**: Starting from a CT-scan DICOM file, we created a 3D-printed liver mold using InVesalius (Centro de Tecnologia da informação Renato Archer CTI, InVesalius 3 open-source software, Campinas, Brazil) for segmentation, Autodesk Fusion 360 with Netfabb (Autodesk software company, Fusion 360 2.0.19426 with Autodesk Netfabb Premium 2023.0 64-Bit Edition, San Francisco, CA, USA) for 3D modeling and Stratasys Fortus 380 mc 3D printer (Stratasys 3D printing company, Fortus 380 mc 3D printer, Minneapolis, MN, USA). Using the 3D-printed mold, we created 14 gelatin-based liver phantoms with 14 different recipes, using water, cast sugar and dehydrated gelatin, 32% fat bovine milk cream with intravenous lipid solution and technical alcohol in different amounts. We tested all these phantoms as well as ex vivo pig liver and human normal, fatty and cirrhotic liver by measuring the elasticity, shear wave speed, ultrasound attenuation, CT-scan density, MRI signal intensity and fracture force. We assessed the results of the testing performed, as well as the optical appearance on ultrasound, CT and MRI, in order to find the best recipe for gelatin-based phantoms for image-guided procedure training. **Results**: After the assessment of all phantom recipes, we selected as the best recipe for transparent phantoms one with 14 g of gelatin/100 mL water and for opaque phantom, the recipes with 25% cream. **Conclusions**: These liver gelatin-based phantom recipes are an inexpensive, reproducible and accessible alternative for training in image-guided and diagnostic procedures and will meet most requirements for valuable training.

## 1. Introduction

Patients with liver pathologies (tumors, abscesses, cystic formations, etc.) can benefit from image-guided interventions. Invasive procedures on the liver are usually performed either following imaging evaluation and/or under imaging guidance.

Ultrasound (US) was used for the first time by surgeons in the USA. The American College of Surgeons pioneered the use of diagnostic ultrasound training for surgeons, with the first postgraduate course in 1996 [1]. Also, the Korean Society of Surgery requested and recommended that all general surgery residents obtain competence in ultrasonography in order to be able to use ultrasonography in surgical interventions [1]. Ultrasound-guided parenchyma-sparing liver resections using intraoperative ultrasound brings a major benefit to patients by minimizing the resected healthy liver tissue [2]. All these procedures, diagnostic and interventional, require the surgeons to train and go through a learning curve until becoming competent enough to perform them. Various national and international organizations require a specific curriculum to be certified, together with a recorded number of supervised procedures performed. In the USA (Gastrointestinal Core Curriculum of the American Gastroenterological Association), a mandatory number of 40 US-guided liver biopsies performed under supervision are required for advanced training in hepatology [3]. In Romania, to obtain the certification in general ultrasonography, it is necessary to perform 300 examinations [4], and for interventional ultrasonography, there is no specific certification and, consequently, no minimum number of procedures that must be performed under the supervision of an expert. In the case of a learning curve for CT-guided procedures, Park R. et al. [5] established that for transthoracic percutaneous CT-guided biopsy, at least 37 procedures are required to achieve acceptable diagnostic accuracy.

Training for interventional procedures (punctures, biopsies, drainage, etc.) is recommended to be performed on liver phantoms until a basic proficiency is reached, and several commercially available phantoms are available for these purposes. Commonly used are Triple modality 3D abdominal phantom model 0557A (CIRS Inc., Norfolk, VA, USA) [6], Abdominal Intraoperative & Laparoscopic Ultrasound Phantom—IOUSFAN, produced by Kyoto Kagaku Co., Ltd. (Kyoto, Japan) [7] and several others. These are available at prices ranging from USD 2897 [8] to USD 8700 [9].

In the last 40 years, attempts have been made to develop materials which mimic the ultrasonic characteristics of human liver in order to create soft liver phantoms for training in US-guided liver procedures. A review of these efforts was published in 2010 by Culjat et al., describing different organ phantoms based on gelatin, agarose, magnesium silicate, oil gel, open-cell foam, polyacrylamide gel, polyurethane gel and organic tissue [10]. In these studies, liver phantoms were uncommon [11,12,13,14], and the majority of them were designed for imaging procedures: for ultrasound [15,16,17], CT [18,19,20,21] and MRI [22,23].

There is a lack of accessible, reproducible and cost-effective anatomically reliable soft liver phantoms for training in image-guided procedures. We developed and validated multiple recipes for gelatin-based liver phantoms dedicated for training in several image-guided procedures as described below.

## 2. Materials and Methods

Our aim was to create an anatomical phantom suitable for the training of young surgeons for multiple targeted, minimally invasive procedures, such as diagnostic ultrasound (US), US elastography, US-guided punctures, biopsy and drainage, and US-guided radiofrequency ablation (RFA) needle insertion, and for CT and MRI diagnosis and image-guided interventions.

To obtain a model as close as possible to the liver anatomy, we created and tested multiple recipes of gelatin-based anatomical soft liver phantoms that are easy to produce, use and store and are inexpensive and based on easily available materials.

Following the completion of the phantoms, we compared the different molds based on a set of characteristics to define the best model which was then used in multiple training sessions.

We produced multiple gelatin-based life-size and human-like soft liver phantoms cast in a modular 3D-printed mold, using different recipes in order to mimic the US, CT and MRI image appearance of the liver. We first performed an accurate segmentation of a liver from a CT-scan DICOM file of a patient using an open-source software—InVesalius (Centro de Tecnologia da informação Renato Archer CTI, InVesalius 3, open-source software, Campinas, Brazil), and obtained a stereolithography (STL) file:

Using the “region growing” function, the liver parenchyma was selected, and the unwanted parts of the model were removed using “crop” and “delete” functions.

From the “surface” menu, the “smooth” function was used for smoothing the surface of the liver model.

From the “surface” menu, the “close holes” function was used for closing the holes in the surface of the liver model.

At the end of the segmentation process, the STL file was generated.

**Figure 1:**
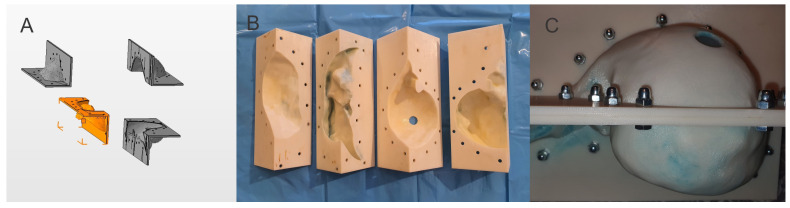
(**A**) Virtual 3D modular liver mold; (**B**) Disassembled 3D-printed liver mold; (**C**) Assembled 3D-printed liver mold.

By 3D modeling using “Autodesk Fusion 360 with Netfabb” software (Autodesk software company, Fusion 360 2.0.19426 with Autodesk Netfabb Premium 2023.0 64-Bit Edition, San Francisco, CA, USA), we created 4 virtual segments that were easy to assemble to form a virtual mold (Figure 1A).

Using the STL file, the first step was to check and correct the 3D model’s possible integrity problems using the “automatic repair” function.

Overlapping geometries were adjusted using the “cut” and “delete” function.

The surface of the liver 3D model was optimized by reducing the number of triangles using the “reduce” function, improving the 3D-printing model’s performance.

A thickness of 5 mm was given to the model’s outer surface by using the “extrude” function, and then, the model was cut in 4 segments using the “cut” function. And then, geometries were created on each segment using again the “extrude” function.

On geometries created on the 4 segments, assembly holes were created using the “Boolean” function, and a casting hole was created on the highest point of the virtual mold using again the “cut” function. At the end of the 3D modeling, the printable STL file was created using the “export” function.

We 3D-printed 5 mm thick mold segments using an FDM technology (fused deposition modeling) 3D printer, Stratasys Fortus 380 mc (Stratasys 3D printing company, Fortus 380 mc 3D printer, Minneapolis, MN, USA) (the 3D printing was performed by Nutechnologies ltd, Timisoara, Romania), resulting in an assembled mold of 300 mm length, 200 mm width and 200 mm height, with an inner volume of 1533.7 cm^3^ (Figure 1B,C), similar to an average healthy adult liver, i.e., between 1445.2 cm^3^ [24] and 1541 cm^3^ [25].

We chose gelatin as the base material from the review article by Culjat MO et al. [10], and for the recipe, we combined the recipe for home-made ballistic gel with cat sugar, a base ingredient for gummy bears. We started from a recipe for a home-made gelatin-based ballistic gel [26], which uses 34.1 L (9 gallons) of water and 3316.89 g (117 ounces) of gelatin, with a dosage of 9.73 g of gelatin per 100 mL of water. We tested 4 recipes with different amounts of gelatin: 8 g, 12 g, 14 g and 16 g of gelatin to every 100 mL of water. In addition, we added different amounts of commercial sugar for strengthening the structural integrity of the phantoms adding for each 100 mL: 8 g gelatin + 12.5 g sugar, 12 g gelatin + 15 g sugar, 14 g gelatin + 17.5 g sugar and 16 g gelatin + 20 g sugar.

**Figure 2:**
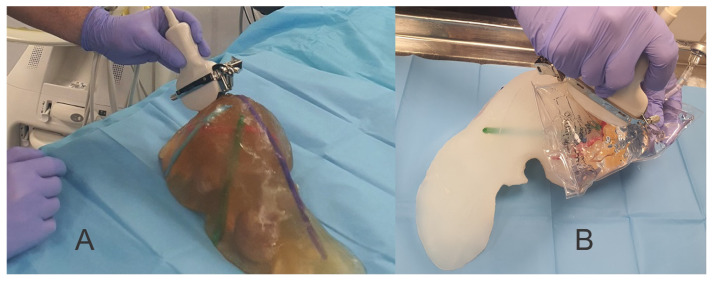
(**A**) Gelatin-based liver phantom; (**B**) Shore-13 silicone liver phantom.

For the validation process, we created phantoms using the 4 recipes with different concentrations of gelatin and sugar as described, and the research team (7 consultant surgeons, 2 specialist surgeons and 2 engineers, with 6 of the surgeons with competence in ultrasonography) made the first analysis to find the proper concentration of gelatin for phantoms to be used in image-guided procedures. For this, we tested the phantoms together with an ex vivo pig liver and a Shore A 13 silicone model (Figure 2).

We defined a set of 9 criteria to assess the 4 gelatin-based recipes developed so far, along with Shore A 13 silicone model compared to ex vivo pig liver:

Criteria 1: hardness—how hard/soft is the model when handling;
Criteria 2: friability—how easy or not the model fracture when it is used for training;
Criteria 3: handling—how easy or not the model can be handled without being damaged;
Criteria 4: optimal characteristics for ultrasound—how optimal/not optimal is the model for ultrasound examination;
Criteria 5: optimal characteristics for elastography—how optimal/not optimal is the model for ultrasound elastography;
Criteria 6: optimal characteristics for Fibroscan—how optimal/not optimal is the model for Fibroscan examination;
Criteria 7: optimal for multiple punctures—how well/not well does the model behave for multiple puncture;
Criteria 8: puncture resistance—how easy/difficult is to puncture the model;
Criteria 9: optimal for training in ultrasound-guided procedures—how optimal/not optimal is the model for ultrasound-guided procedures.

Based on these criteria, the phantom recipes were evaluated using a 5-point Likert scale (1:5), with the following values: 1—very poor performance, 2—mediocre performance, 3—acceptable performance, 4—good performance, 5—very good performance (Table 1).

After the analysis, we identified that the concentration of 14 g of gelatin per 100 mL of liquid gives the model the closest characteristics to those of ex vivo pig liver for US-guided procedures. Thus, the model with 14 g, as well as the one with 16 g of gelatin, was proven to be comparable to be handled as a fresh pig liver and offered suitable characteristics for US examination and elastography, allowing multiple US-guided punctures, as well as preservation of resistance over 5 weeks of use if the models are kept at 0–4 °C. The model with 16 g of gelatin was found to have increased rigidity, friability and puncture resistance compared to the model with 14 g of gelatin (much like a cirrhotic liver). After this first evaluation, we found the 14 g of gelatin/100 mL liquid to be the best basic recipe for gelatin-based liver phantoms.

For increasing the speed of sound into the material we use technical alcohol (with >90% concentration of technical ethyl alcohol and isopropyl alcohol) to replace 10% or 20% of the water amount.

**Figure 3:**
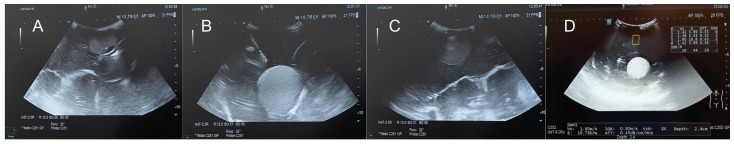
(**A**) Corn starch; (**B**) Wheat flour; (**C**) Talcum powder; (**D**) 32% bovine milk fat/i.v. lipid solution.

For contrast from surrounding tissue and for the scattering effect, we tested multiple substances: corn starch, wheat flour, talcum powder and a lipid solution (lipid solution for intravenous administration) at 5%, 10% and 15% from the liquid amount and commercial milk cream (32% fat) with 25% of the entire liquid amount (Figure 3).

The research team (7 consultant surgeons, 2 specialist surgeons and 2 engineers, with 6 of the surgeons with competence in ultrasonography) conducted the analysis of these substances to find the proper one to be used for a scattering effect and as a contrast for tumors in transparent phantoms. For that, we defined a set of 4 criteria to assess the wheat flour, corn starch, talcum powder and 32% bovine milk fat:

Criteria 1: contrast to the surrounding tissues—how good/poor is the contrast to the surrounding tissues.
Criteria 2: visible limit compared to the surrounding tissues—how easy/difficult is to identify the limit between the contrast and non-contrast tissue.
Criteria 3: easy to identify from the surrounding tissues (even at small sizes)—how easy/difficult is to identify a contrast tissue inside the model.
Criteria 4: homogeneity—how homogeneous is the gelatin-based contrast solution.

Based on these criteria, the 4 substances were evaluated using a 5-point Likert scale (1:5), with the following values: 1—very poor performance, 2—mediocre performance, 3—acceptable performance, 4—good performance, 5—very good performance (Table 2).

To lower the density of the material, we reduced the amount of sugar added into the phantom where we used 25% milk cream. We found talcum powder, corn starch and wheat flour inappropriate because they did not create a homogeneous composition due to their settling during solidification (Figure 3).

**Figure 4:**
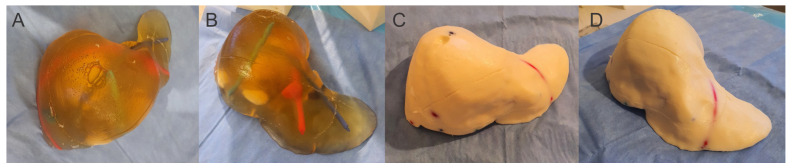
(**A**,**B**) Transparent liver phantom; (**C**,**D**) Opaque liver phantom.

For the final testing, we created 14 different gelatin-based recipes for liver phantoms for training in image-guided procedures. Six of them were transparent (G8, G12, G14, G16, G14alc 10, G14alc 20), and the rest were opaque (Figure 4), as follows:

G8: 8 g gelatin/100 mL liquid (water) + 12.5 g sugar
G12: 12 g gelatin/100 mL liquid (water) + 15 g sugar
G14: 14 g gelatin/100 mL liquid (water) + 17.5 g sugar
G16: 16 g gelatin/100 mL liquid (water) + 20 g sugar
G14i5: 14 g gelatin/100 mL liquid (95% water, 5% intravenous lipid solution) + 17.5 g sugar
G14i10: 14 g gelatin/100 mL liquid (90% water, 10% intravenous lipid solution) + 17.5 g sugar
G14i15: 14 g gelatin/100 mL liquid (85% water, 15% intravenous lipid solution) + 17.5 g sugar
G14alc10: 14 g gelatin/100 mL liquid (90% water, 10% technic alcohol) + 17.5 g sugar
G14alc20: 14 g gelatin/100 mL liquid (80% water, 20% technic alcohol) + 17.5 g sugar
G14s32:17.5: 14 g gelatin/100 mL liquid (75% water, 25% cream solution) + 17.5 g sugar
G14s32:15: 14 g gelatin/100 mL liquid (75% water, 25% cream solution) + 15 g sugar
G14s32:12.5: 14 g gelatin/100 mL liquid (75% water, 25% cream solution) + 12.5 g sugar
G14s32:10: 14 g gelatin/100 mL liquid (75% water, 25% cream solution) + 10 g sugar
G14s32:7.5: 14 g gelatin/100 mL liquid (75% water, 25% cream solution) + 7.5 g sugar

We have tested all 14 gelatin-based phantom recipes together with an ex vivo pig liver using ultrasound, CT-scan and MRI, measuring elasticity (kilopascal—kPa), ultrasound attenuation (decibels/centimeter/megahertz—dB/cm/MHz), shear wave speed (meters/second—m/s) using a Hitachi Arietta 850 ultrasound machine (Hitachi, FujiFilm Healtcare Corporation, Chiba, Japan), CT-scan density (Hounsfield unit—HU) using a 128 slice Siemens CT scan (Somatom Definition Edge SW VA48, Siemens Healtcare GmbH, Eriangen, Germany), MRI signal intensity (arbitrary units—a.u.) using a 1.5 Testa General Electric MRI (SIGNA Explorer 1,5T, GE Healthcare Company Limited, Tianjin, China), and fracture force (kilonewton—kN) measured by pressing the models with an electronic press (WEW-600D Universal Testing Machine, No. 8003/2016.3, TIME Group Inc., Shangdi Industrial Base, Beijng, China, last calibration on 13 March 2023). All the examinations were performed on the same phantom recipe models, included the ex vivo pig liver, and all the measurements were performed as follows:

Elasticity, ultrasound attenuation, shear wave speed and CT-scan density were performed 20 times.

MRI signal intensity (SI) was measured 10 times for T1 and 10 for T2.

Fracture force was measured once on each model because the model was damaged during the procedure.

We proceeded to compare the results of the liver phantom recipe tests with those of pig liver (ex vivo) and with those of real patients (human liver), examined by us and with data from the literature, in order to find out which gelatin-based liver phantom recipe is most suited for training in different image-guided or diagnostic procedures. And we analyzed the optical appearance of the phantom recipes in different imaging examinations. We also measured the time needed for casting one or multiple liver phantoms using one or multiple molds.

## 3. Results

We casted the 14 different recipes of gelatin-based phantoms. The median value of elasticity, ultrasound attenuation, shear wave speed, CT-scan density, MRI signal intensity and fracture force value of the phantoms, pig liver and normal, fatty and cirrhotic human livers are listed in Table 3.

We performed the assessment of phantoms in two separate categories: transparent phantoms (G8, G12, G14, G1, G14alc10, G14alc20) and opaque phantoms (the other 8 recipes).

We found G14 to be the least friable of the transparent phantoms’ recipes. All other transparent phantoms were too friable to be used for image-guided procedure training. G14 had the elasticity and shear wave speed values between fatty and cirrhotic liver, with the CT-scan density much higher than a cirrhotic liver and a very high MRI signal intensity. For the first difficulty level in the training in image-guided procedures, G14 is the recommended phantom recipe, with the advantage of being able to see the structures inside (tumors, vascular structures) both by looking at the phantom and on the screen of the imaging device.

For the second difficulty level in the training in image-guided procedures, we recommend the use of opaque phantoms to be able to identify the structures inside by imaging only.

Analyzing the characteristics of the opaque phantom recipes, we found that the elasticity of all phantoms with lipid content as well as that of ex vivo pig liver corresponded to the elasticity of cirrhotic liver.

The ultrasound attenuation of the phantoms with 25% cream solution (32% fat) and lower sugar content (G14s32:15, G14s32:12.5, G14s32:10, G14s32:7.5) were similar to the fatty liver.

The shear wave speed of all the opaque phantoms was similar to a cirrhotic liver.

For CT-scan density, the values of phantoms with intravenous lipid solution (G14i5, G14i10, G14i15), G14S32:17.5 and the ex vivo pig liver were much higher than the fatty and cirrhotic liver. Recipes G14S32:15 and G14S32:12.5 had values of CT-scan density similar to the higher value in the literature (64 [30]), and G14S32:10 and G14S32:7.5 had density similar to the cirrhotic and normal liver according to our evaluation and to the literature: 42–58.32 [31,32] for normal liver and 50.59 [34] for cirrhotic liver, and their values are between the lower and highest values in the literature for fatty liver (32.44–64 [31,32]).

We also found that for phantoms’ recipes with intravenous lipid solution, the higher the amount of fat, the lower was the CT-scan density. For the same amount of fat solution (phantoms with 25% cream solution), the lower the amount of sugar, the lower was the CT-scan density.

The MRI signal intensity of all opaque phantoms was found much higher than normal, fatty or cirrhotic human liver. The ex vivo pig liver had an MRI SI in T2 similar to cirrhotic liver and in T1, similar to G14S32:10, much higher than human liver.

Fracture force similar to the pig liver (1.26 kN) was found at G14S32:17.5 (1.13 kN) and was much higher compared with ex vivo pig liver, as the amount of sugar was lower: +59.52% for G14S32:15, +71.43% for G14S32:12.5, +80.95% for G14S32:10 and +43.65% for G14S32:7.5. According to this evaluation, the less friable and the much easier to manipulate and to work with are the G14S32:12.5 and G14S32:10 gelatin-based liver phantom recipes.

According to the analysis of the basic gelatin solution recipes (G8, G12, G14, G16), the optimal amount of gelatin for liver phantoms is 14 g/100 mL solution, with an elasticity of 13 kPa, close to a cirrhotic liver (14 kPa [24]) and a fracture force of 44.7% from the pig liver fracture force, which makes this recipe the most elastic and least friable among the basic transparent recipes. This is the best recipe for transparent phantoms. We consider this the basic recipe for all other gelatin-based liver phantoms.

Based on the assessment of the optical appearance of the phantom recipes in different imaging examinations, we select the phantom recipe which is suited to be used in diagnostic and image-guided procedures and which are closer to the real normal, fatty and cirrhotic human liver appearance on ultrasound, CT-scan evaluation and MRI (Appendix A).

A separate assessment was performed to optimize simulated tumors inside the phantom. We used different recipes: for the transparent phantoms, we used opaque tumors using the G14S32:15 recipe, and for opaque phantoms, we used transparent tumors using the G14 gelatin-based recipe.

The best optical appearance of the transparent phantoms ware G14 and G16 recipes. But because the G16 had a lower fracture force, we recommend the G14 gelatin-based phantoms recipe to be used in training for image-guided procedures and diagnostic.

**Figure 5:**
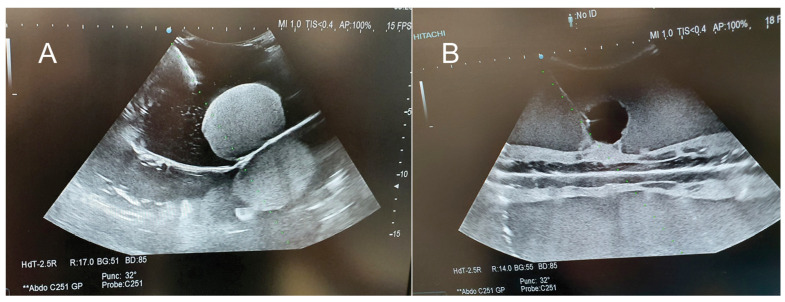
(**A**) Parenchyma G14 and tumor G14S32:15; (**B**) Parenchyma G14S32:12.5 and tumor G14.

Opaque phantom gelatin-based recipes with the best optical appearance for training in image-guided procedures and diagnostic are G14S32:15, G14S32:12.5 and G14S32:10, because in ultrasound, they produce a homogenous parenchyma and a uniform scattering effect with a good contrast on CT-scan and MRI T1 and a very good contrast in ultrasound (Figure 5) and MRI T2. These optical characteristics are similar in G14i5, G14i10 and G14i15, but these phantoms have a much lower fracture force value which makes them more friable.

The average needed to cast one gelatin-based liver phantom with vascular structures and tumor formations inside one mold is 100–110 min.

The cost of the liver phantoms using these recipes after the mold has been produced may vary between 10 and less than 20 euro, which makes them an inexpensive alternative for training in image-guided and diagnostic procedures. The cost of every phantom may vary because of daily cost of materials (Table 4).

## 4. Discussion

Until now, multiple solutions have been devised to mimic liver parenchyma. We decided to create real-size liver shape phantoms cast in 3D-printed modular molds using a gelatin-based solution. This solution to mimic the liver tissue for ultrasound examination training was first described by Burlew et al. and Ophir et al. [37,38,39]. There are many other solutions described for mimicking the liver parenchyma, like agarose-based (Madsen et al., 1998, 2003; Burlew et al., 1980; D’Souza et al., 2001; Ramnarine et al., 2001; Brewin et al., 2008) [38,40,41,42,43,44], magnesium silicate-based (Sheppard and Duck 1982) [45], oil gel-based (mixture of propylene-glycol, dibenzylidene D-sorbitol (gelatinizer) and polymethyl methacrylate mycrospheres) (Kondo et al., 2005; Dong et al., 1999) [46,47], open-cell foam-based (polyurethane foam and salt (NaCl) water) (Ophir 1981, 1984; Lerski et al., 1982) [39,48,49], polyacrylamide gel-based (polymerization of the acrylamide monomer + water) (Zell et al., 2007) [50], polyurethane gel-based (Kondo et al., 2005), polyvinyl alcohol-based (polyvinyl alcohol 10% weight + water) (Fromageau et al., 2003; Kharine et al., 2003; Surry et al., 2004) [51,52,53], silicone polymer-based (silicone polymer) (Robertson et al., 1992; ICRU 1998) [54,55] and organic-based (tofu, porcine and bovine liver) (Wojcik et al., 1999; Kim et al., 2009; Davies and Kew 2001; XU et al., 2005) [56,57,58,59]. The most common solutions for creating liver phantoms are the gelatin-based (with graphite powder) and the open-cell foam with water [10]. Breast phantoms have also been used to mimic liver parenchyma for training in ultrasound-guided biopsies [60].

The most affordable solution to mimic the liver tissue to create phantoms for image-guided procedures and diagnostics is the gelatin-based solution. Also, this is easy to be produced, and it may take less than 90 min for one real-size liver-shaped gelatin-based phantom to be cast. When not used and stored at 0–4 °C, it can last for more than a month.

In 1995, Bude and Alder described a recipe of an opaque and clear gelatin-based phantom for ultrasound examination with “mases” (carrot or hot-dog pieces, olives, macaroni) and “cysts” (balloons, grapes, tips of examining gloves), using 20 g of unflavored gelatin for 250 mL of water, and for the scattering effect, they used psyllium hydrophilic mucilloid fiber [61]. They also stated that if refrigerated, these gelatin-based phantoms can be stored several weeks without significant microbial degeneration [61].

For the scattering effect and attenuation, we first used wheat flour, corn starch and talcum powder, used by Sheppard and Duck in 1982 [45], for a magnesium silicate-based phantom. All these powders tend to settle because they do not dissolve in the gelatin-based solution. Secondly, we used intravenous lipid solution and cream solution with 32% fat, which are emulsified fats and do not settle when mixed with the gelatin solution, also described by Rethy A et al. [62]. To optimize the scattering effect and attenuation, researchers also used evaporated milk which can easily dissolve in water solution [38,40,41,42,43,44], graphite powder [37,38,39,40,41,42,43,44,45] and glass or plastic microspheres [51,52,53,54,55,56,57,58,59].

Recipes designed by our team with 5%, 10% and 15% intravenous lipid solution (G14i5, G14i10, G14i15) have elasticity similar to human cirrhotic liver and to ex vivo pig liver and shear wave speed similar to human fatty liver and cirrhotic liver and to ex vivo pig liver, which makes these recipes valuable for opaque liver phantoms even if the fracture force and CT-scan density are similar to the G14 recipe. Recipes with 25% cream solution with 32% fat with 17.5 g or less amount of sugar/100 mL gelatin solution (G14S32:15, G14S32:12.5, G14S32:10, G14S32:7.5) have elasticity similar to cirrhotic liver and ex vivo pig liver, ultrasound attenuation similar to ex vivo pig liver and human fatty liver, shear wave speed similar to ex vivo pig liver and human fatty and cirrhotic liver, CT-scan density similar to ex vivo pig liver and human fatty and cirrhotic liver and fracture force 43.65% to 80.95% higher than that of ex vivo pig liver (which means these recipes are the lees friable of all gelatin-based liver phantom recipes). All these characteristics make these recipes the most valuable for making opaque liver phantoms for training in ultrasound, CT-scan and MRI diagnostic and interventional procedures [63,64,65].

For increasing the speed of sound, we used technical alcohol with over 90% isopropyl alcohol and ethanol. For the same purpose, n-propanol was used [37,38,39,40,41,42,43,44,45]. By adding 10% or 20% technical alcohol (G14alc10, G14alc20), we only increased the shear wave speed through the phantom, and a CT-scan density was seen similar to human cirrhotic liver, but not more compared with the phantoms with lipid solution in the recipe.

For hardening the phantom gelatin-based solution, we used different amounts of cast sugar. The less amount of sugar we used, the less friable and with a lower CT-scan density was the phantom. In the case of magnesium silicate—based phantom (Sheppard and Duck 1982) [45] used tetrasodium pyrophosphate for hardening of the gel.

We did not use any preservatives for our phantom recipes, although solutions like p-methyl/n-propyl benzoic acid [37,38,39] and thimerosal [38,40,41,42,43,44] were described, and since our phantoms were very cheap to produce and could be stored in a commercial refrigerator, we found no reason to add any additional components.

The cost of our phantoms is between 10 and 13 euro for basic transparent phantom with only gelatin-based “tumors” and balloons as vascular structures inside and 17–20 euro for the opaque phantom with 25% amount of bovine milk cream with 32% fat and many “tumors” and “vascular structures” inside of various materials. The price can vary between these values because of the daily prices of ingredients and the complexity of the phantom, but the highest price does not exceed 20 euros for one phantom. In the literature, there are not many papers where the actual cost of the phantoms has been published. Shrimal P et al. made low-cost gelatin-based phantoms for ultrasound-guided needle tracking for USD 6–8, compared to USD 1000–12,000 for commercial ones [66]. Also Richardson C et al. developed low-cost gelatin-based phantoms for training in ultrasound-guided head and neck procedures for less than USD 20 for each model [67]. The commercial liver phantoms available on the market are far more expensive with prices ranging between USD 2897 and USD 8700 [8,9].

## 5. Conclusions

After assessment of all the gelatin-based liver phantom recipes, we found the optimal recipe to be used for training in image-guided and diagnostic procedures. We recommend using the transparent phantom G14 recipe for beginners and the opaque G14S32:12.5 and G14S32:10 recipes for more advanced trainees. These liver gelatin-based phantom recipes are an inexpensive, reproducible and accessible alternative for training in image-guided and diagnostic procedures and will meet most requirements for valuable training.

## Figures and Tables

**Table 1:** First assessment of liver phantom recipes.

	Criteria	Ex Vivo Pig Liver	Silicone Liver Shore A 13	Gelatin Liver—16 g	Gelatin Liver—14 g	Gelatin Liver—12 g	Gelatin Liver—8 g
1	Hardness	3	5	4	4	3	2
2	Friability	3	1	4	3	4	5
3	Handling	4	5	3	4	3	1
4	Optimal characteristics for ultrasound	5	2	3	4	3	2
5	Optimal characteristics for elastography	5	2	3	4	4	2
6	Optimal characteristics for Fibriscan	5	0	0	0	3	0
7	Optimal for multiple punctures	4	5	3	4	3	2
8	Resistance to punctures	3	5	4	3	2	2
9	Optimal for training in US-guided procedure	5	2	4	5	4	2
	Total	37	27	28	31	29	18

**Table 2:** Assessment of substances for contrast and scattering effect.

	Criteria	Wheat Flour	Corn Starch	Talcum Powder	32% Fat Bovine Milk/i.v. Lipid Solution
1	Contrast to the surrounding tissues	4	3	2	5
2	Visible limit compared to the surrounding tissues	5	3	3	5
3	Easy to identify from the surrounding tissues (even at small sizes)	5	3	2	5
4	Homogeneity	3	2	2	5
	Total	17	11	9	20

**Table 3:** Evaluation of the gelatin-based liver phantom recipes, pig liver and normal, fatty and cirrhotic human liver.

	Elasticity (Kilopascal—kPa)	Ultrasounds Attenuation (dB/cm/MHz)	Shear Wave Speed (m/s)	CT-Scan Density (Hounsfield Unit—HU)	MRI–T1 Signal Intensity (SI-a.u.)	MRI–T2 Signal Intensity (SI-a.u.)	Fracture Force (Kilonewton—kN)
G8	7.67	0.04	1.60	52.29	1012.7	1355.4	0.23
G12	11	0.10	1.9	71	1329.1	808.5	0.30
G14	13	0.09	2.08	82.07	1458.1	1103.3	0.55
G16	5.84	0.12	1.39	92.32	1607.6	890.1	0.38
G14i5	30.9	0.15	3.21	83.25	1489.4	1642.7	0.50
G14i10	33	0.20	3.32	80.73	1547.6	1289.6	0.52
G14i15	39.2	0.28	3.62	71.5	1223.6	1863.7	0.67
G14alc10	14.9	0.08	2.23	70.75	917.5	2162.3	0.38
G14alc20	23	0.11	2.77	60.43	1054.4	1904.5	0.38
G14s32:17.5	38.3	0.93	3.57	74.87	1025.6	1574.6	1.13
G14s32:15	32.7	0.71	3.30	65.69	993.3	1414.6	2.01
G14s32:12.5	35.45	0.71	3.44	64.75	1333.6	1336.2	2.16
G14s32:10	27.1	0.75	3	50.85	1144.6	1314.8	2.28
G14s32:7.5	35.15	0.67	3.42	49.9	1398.8	1397.4	1.81
Pig liver (ex vivo)	32.1	0.94	3.27	76.06	1131.5	176.1	1.26
Human normal liver	4.5 4.8 [27] 4.93 [28]	0.5 0.552 ± 0.03 [29] 0.5 (normal)–1.1 (severe steatosis) [30]	1.23 1.3 [27]	51.02 42 [31]–58.32 [32]	402.1	215.4	N.A.
Human fatty liver (with no fibrosis)	4.47 No significant difference compared to normal liver [33]	0.56 0.69 [34]	1.22 3.42 [34]	36.1 32.44 [32]–64 [32]	453.83	282.7	N.A.
Human cirrhotic liver	35.8 14 [27] 25.8 [35] 27.5–62.7 [36] 13.29 [28]	0.5 0.58 [34]	3.45 2.2 [27] 2.61 [34]	50.46 50.59 [32]	363.38	188.95	N.A.

N.A.: not applicable, a.u.: arbitrary units.

**Table 4:** Average cost of gelatin-based phantoms created by our team.

	G8 G12	G14 G16	G14i5 G14i10 G14i15	G14alc10 G14alc20	G14s32:7.5 G14s32:10 G14s32:12.5	G14s32:15 G14s32:17.5
Cost (euro)	10–11	11–12	11–14	12–13	14–17	17–19

## Data Availability

The data presented in this study are available on request from the corresponding author (graurf@yahoo.com).

## Appendix A

**Table A1:** The gelatin-based liver phantom recipes compared to ex vivo pig liver and real human liver image assessment and their fracture force graphic where applicable.

	G8	G12	G14
Ultrasound			
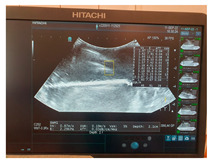
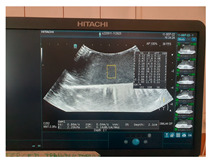
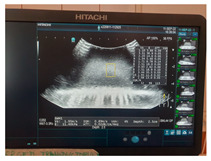
CT-scan			
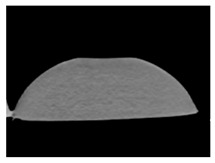
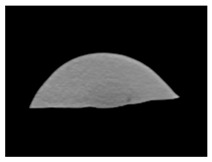
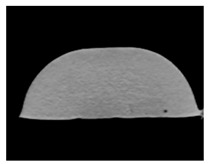
MRI–T1			
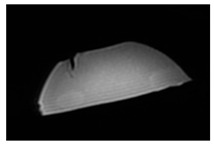
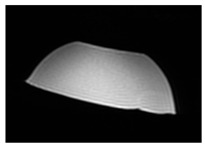
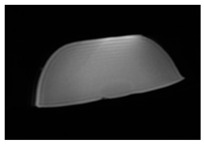
MRI–T2			
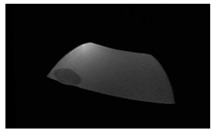
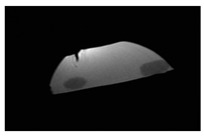
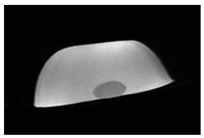
Fracture force graphic			
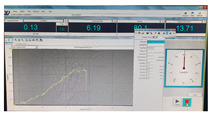
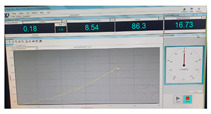
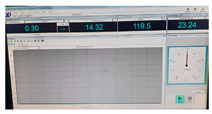
	G16	G14Alc10	G14Alc20
Ultrasound			
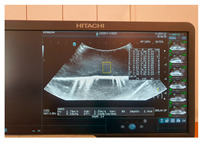
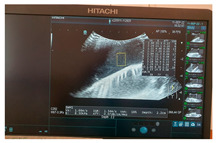
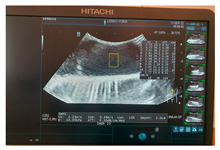
CT-scan			
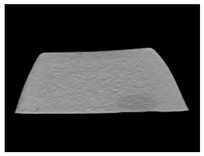
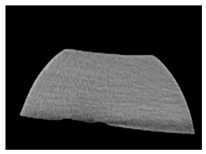
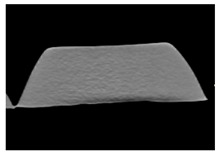
MRI–T1			
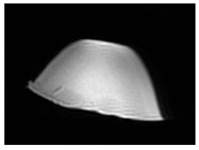
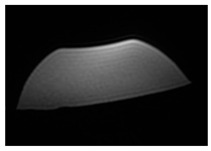
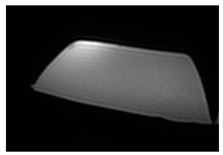
MRI–T2			
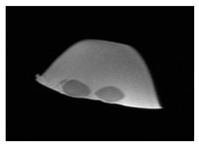
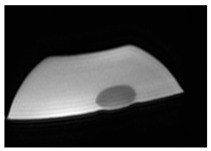
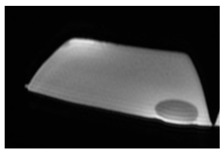
Fracture force graphic			
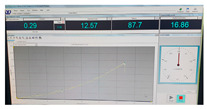
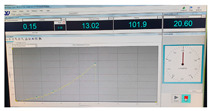
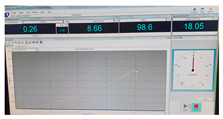
	G14i5	G14i10	G14i15
Ultrasound			
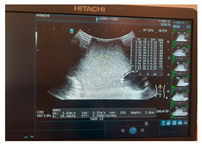
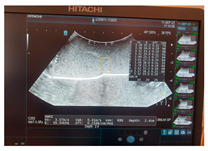
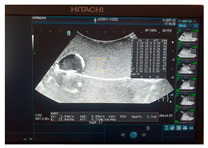
CT-scan			
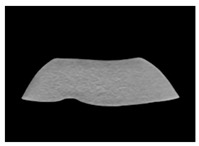
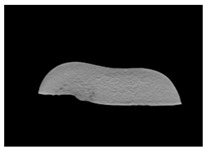
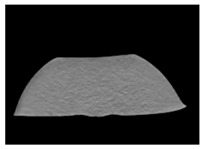
MRI–T1			
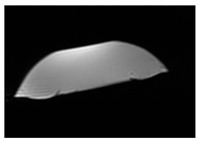
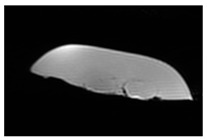
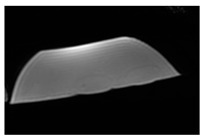
MRI–T2			
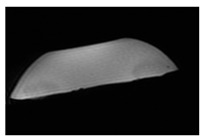
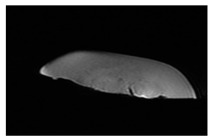
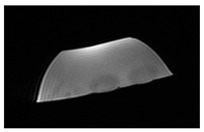
Fracture force graphic			
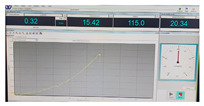
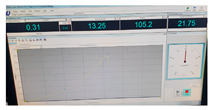
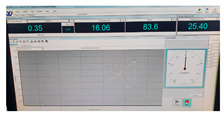
	G14S32:25-17.5	G14S32:25-15	G14S32:25-12.5
Ultrasound			
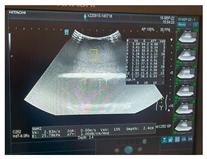
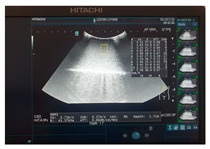
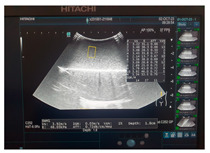
CT-scan			
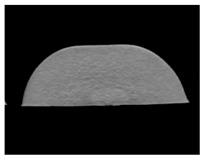
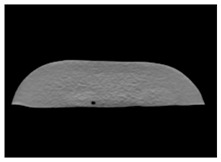
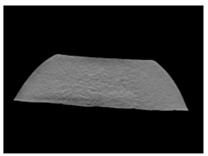
MRI–T1			
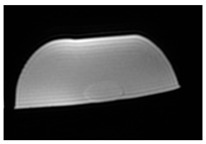
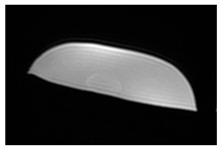
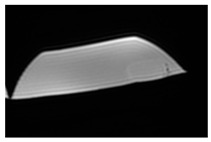
MRI–T2			
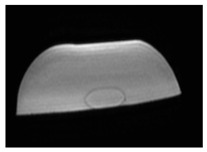
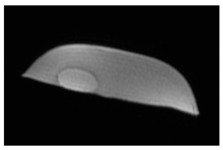
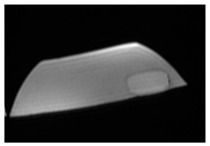
Fracture force graphic			
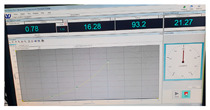
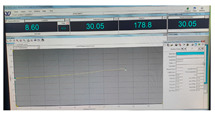
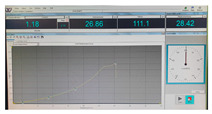
	G14S32:25-10	G14S32:25-7.5	Pig liver (ex vivo)
Ultrasound			
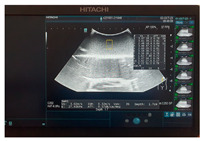
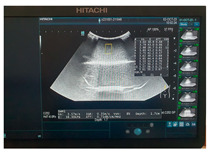
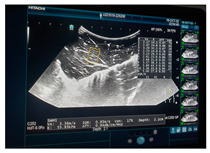
CT-scan			
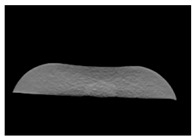
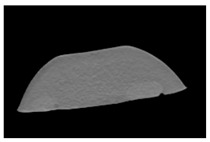
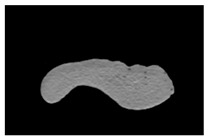
MRI–T1			
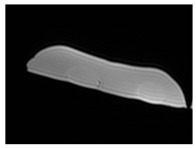
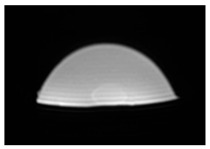
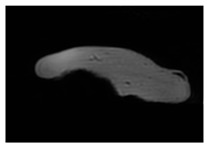
MRI–T2			
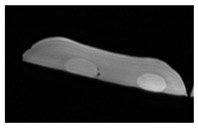
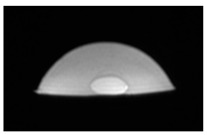
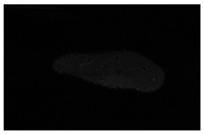
Fracture force graphic			
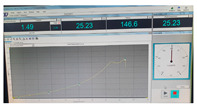
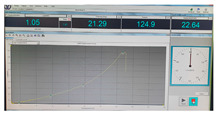
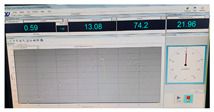
	Human normal liver	Human fatty liver	Human cirrhotic liver
Ultrasound			
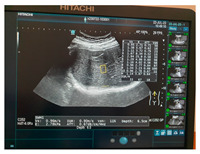
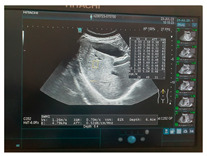
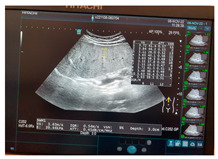
CT-scan			
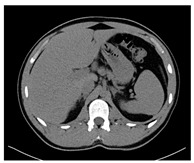
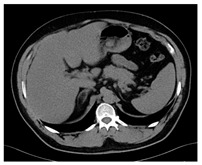
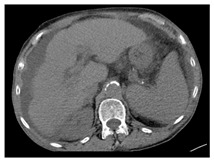
MRI–T1			
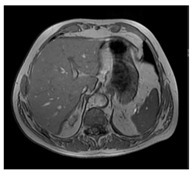
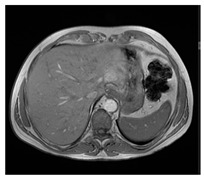
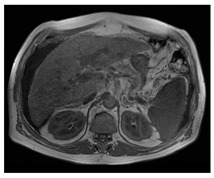
MRI–T2			
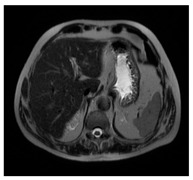
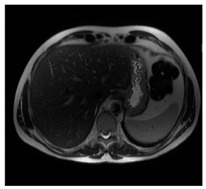
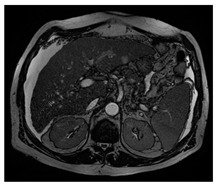
Fracture force graphic	N.A.	N.A.	N.A.

N.A.: not applicable.
